# Sports participation in childhood and adolescence and physical activity intensity in adulthood

**DOI:** 10.1371/journal.pone.0299604

**Published:** 2024-05-02

**Authors:** Mariana Biagi Batista, Mileny Caroline Menezes de Freitas, Catiana Leila Possamai Romanzini, Cynthia Correa Lopes Barbosa, Gabriela Blasquez Shigaki, Rômulo Araújo Fernandes, Marcelo Romanzini, Enio Ricardo Vaz Ronque

**Affiliations:** 1 School of Education, Federal University of Mato Grosso do Sul (UFMS), Campo Grande, Mato Grosso do Sul, Brazil; 2 Laboratory of Physical Activity and Health, Center of Physical Education and Sports, Londrina State University (UEL), Londrina, Paraná, Brazil; 3 Academic Department of Humanities, Federal University of Technology Paraná (UTFPR), Apucarana, Paraná, Brazil; 4 Department of Physical Education, University Paulista (UNIP), São Paulo, Brazil; 5 University Center of Rio Preto (UNIRP), São José do Rio Preto, São Paulo, Brazil; 6 São Paulo State University (UNESP), Presidente Prudente, São Paulo, Brazil; Universiti Malaya, MALAYSIA

## Abstract

**Objectives:**

The aim of the present study was to analyze the association between sports participation in childhood and adolescence and the practice of physical activity at different intensities in adulthood, and to verify if some sports participation characteristics such as number of sports; type of sport (individual, collective or a combination of both) and total estimated sports participation time are associated with the different physical activity intensities in adulthood.

**Design:**

This is a cross-sectional study.

**Methods:**

This study included 129 young adults of both sexes aged 18–25 years. Sports participation in childhood (7–10 years) and adolescence (11–17 years) was retrospectively estimated through specific questionnaire. Light, moderate, vigorous and moderate to vigorous intensity physical activity was objectively estimated by accelerometers. To verify the association between SP in childhood and adolescence and BP intensities in adults, multiple linear regression was adopted, with 5% significance.

**Results:**

Analyses showed that, in females, sports participation in childhood (β = 0.315; R^2^ = 0.14; P = 0.020) and persistence in sports participation (β = 0.364; R^2^ = 0.18; P = 0.007) were positive predictors of vigorous physical activity in adulthood. In addition, the comparison according to the specificities of the sport practice, indicated that participation in two or more sports in childhood, one sport and collective sports in adolescence and at least one year of sports participation throughout childhood and adolescence were associated with longer time in vigorous physical activity intensity and MVPA (minutes/day) in adult females (P < 0.05).

**Conclusions:**

It could be concluded that sports participation indicators in childhood and adolescence were considered predictors of vigorous physical activity in adult females. In addition, number of sports, type of sport and practice time in childhood and adolescence seem to predict vigorous and moderate to vigorous levels of physical activity for adult females.

## Introduction

Sports participation (SP) has been associated with improvements in physical health, social interaction and academic performance aspects among pediatric groups [1–4]. Moreover, SP in childhood and adolescence affects physical activity (PA) patterns throughout life, being positively associated with general levels of PA in youth [5–7].

In conceptual terms, PA refers to any bodily movement generated by skeletal muscles that results in energy expenditure above resting levels and manifests in various domains (occupational, commuting, household, and leisure) [8]. Sport is a subset of PA, with defined rules and objectives, individually practiced or in team sports [9]. Thus, SP has been defined as the engagement in organized sports activities, whether in clubs, training schools, or during school extracurricular programs, with the aim of competition or leisure [10].

PA correlates in adulthood have been widely investigated in the last decades. Early PA participation is pointed out as a relevant correlate [11–13], while SP in early life has not been the specific focus in most of these studies, lacking many data on the subject (requirement of individual, collective or ball skills, number of sports, practice time and sex particularities) [7]. Thus, up to the present moment, it can be considered that participation in a variety of sports should be encouraged, as it has been demonstrated that engaging in multiple sports during adolescence is positively associated with moderate to vigorous exercise in adulthood [13], without a clear definition of the specific number of sports to be practiced in youth.

Moreover, most of these studies investigating SP in childhood and adolescence as predictor of PA in adulthood were carried out in developed countries (Finland [14–16], Norway [17], Ireland [5], Australia [18], Sweden [19], Canada [20]) and the inference of their findings in developing countries is not clear, mainly because SP differs in terms of prevalence and preferences around the world [21]. Specifically in the case of Brazil, a similar study investigated the tracking of PA from adolescence to adulthood, using the practice of sports in training clubs and schools, as well as other unsupervised activities like running and walking, as indicators of physical activity during youth [22]. To date, only one study has sought to specifically examine the association between childhood and adolescence SP and different PA intensities in adulthood [23]. Both studies [22, 23] reported positive results regarding the relationship between early-age SP and adult PA levels, but they did not explore specific SP characteristics during childhood and adolescence.

Another gap in literature, which investigated the relationship between SP in childhood and adolescence and PA in adulthood, is the fact that PA has been mostly estimated by subjective methods. Studies that have used objective methods to measure physical activity in adulthood (accelerometry) are still scarce [23, 24, 25], and among these, many did not include all physical activity intensities in their analyses, with a focus on moderate to vigorous physical activity (MVPA) [25, 26]. Furthermore, results have shown gender differences, with studies demonstrating that SP during youth is more strongly associated with PA in adulthood for females [25, 26]. Thus, investigations prioritizing the use of accelerometers for assessing PA should be conducted, considering the analysis of different PA intensities in adulthood, in various populations and contexts in order to clarify the remaining gaps in research addressing this topic.

In this regard, given the current and alarming epidemic of physical inactivity affecting both young people and adults worldwide [35], and considering that physical education (PE) has been identified by the International Society for Physical Activity and Health as an “investment that works” to promote physical activity [27], a better understanding of behaviors related to PE in youth can represent a strategy to combat this serious public health issue.

Thus, the aims of the present study were to analyze the association between SP in childhood and adolescence and PA at different intensities in adult life. Additionally, it compared the time in different PA intensities in adulthood according to SP characteristics (number of sports; type of sport [individual, collective or a combination of both]; total time).

## Materials and methods

### Sample and design

**Fig 1:**
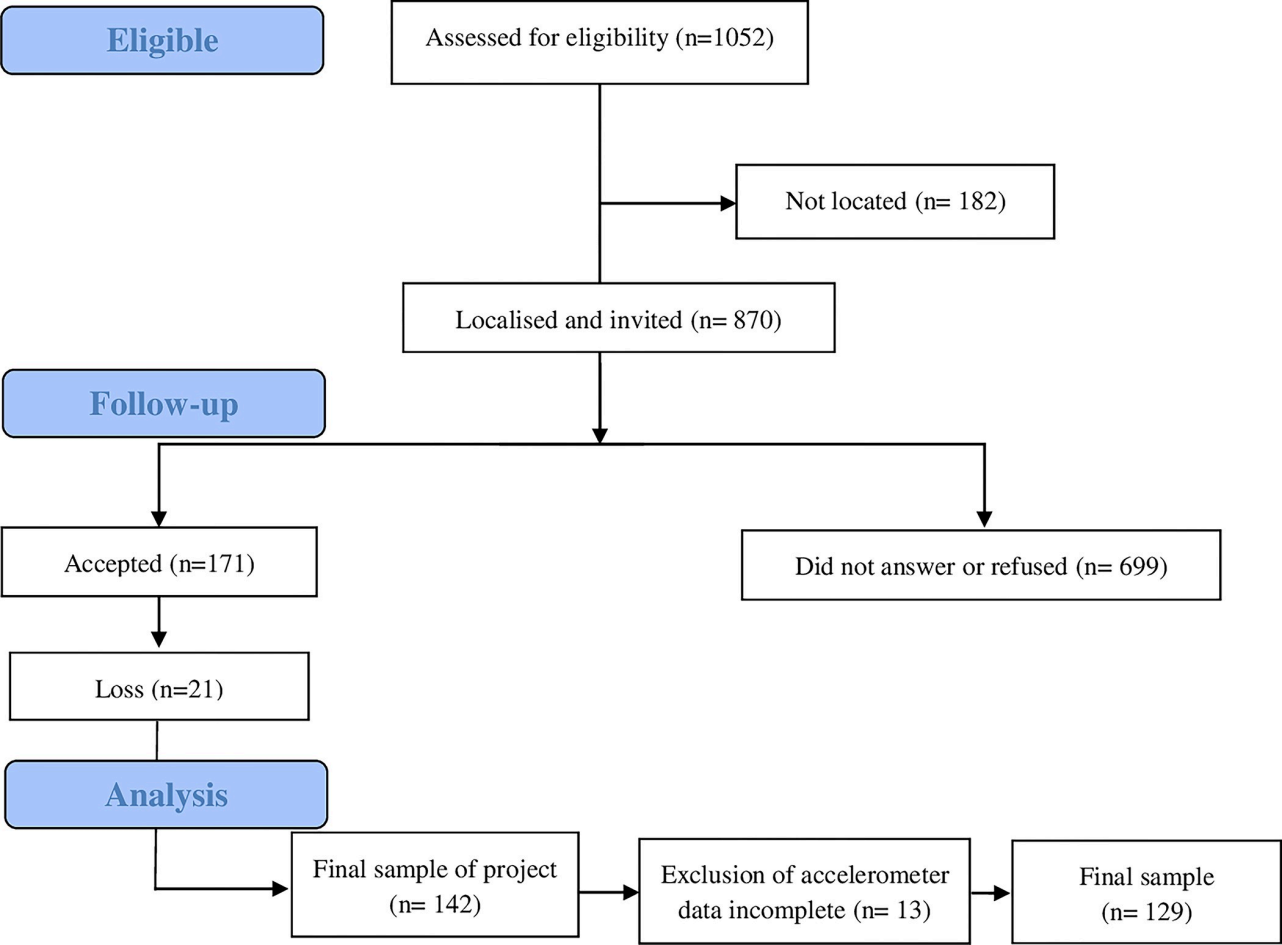
Sample selection consort flow diagram (adapted from Shigaki et al. [29]).

The study included 129 young adults (65 males and 64 females) aged 18–25 years, participants of a mixed longitudinal study carried out from 2002 to 2006 [29]. Sample size was estimated based on the application of linear regression models, with very large effect size and four variables (SP childhood; SP adolescence; persistence in SP and PA adulthood), alfa 0.01 and power of 0.9. For that, the equation 104 + k (k = number of predictors) was used to test each predictor individually [28]. Thus, considering SP in childhood, SP in adolescence and persistence in SP in childhood and adolescence as predictor, sample size of 107 subjects was estimated. Based on the personal information of participants in databases, 1052 subjects were eligible to participate in the study [29]. Therefore, according to the exclusion criteria (valid accelerometry data), the final sample of the present study had 129 participants, described in Fig 1. Participants were informed about the study objectives and procedures to which they would be submitted and signed the Free and Informed Consent Form. The research was approved by the Research Ethics Committee of the State University of Londrina, according to rules of Resolution 466/2012 of the National Health Council on research involving human beings, under protocol No. 1.340.735 of 11/27/2015.

### Sports participation (SP) in childhood and adolescence

Information regarding SP during childhood (7–10 years) and adolescence (11–17 years) was obtained by means of a retrospective instrument validated for population [30] using the following questions: “*Outside school*, *have you been involved in any organized and supervised sports activities for at least 1 year*, *during the time you were 7–10 years old*?” and “*Outside school*, *have you been involved in any organized and supervised sports activities for at least 1 year*, *during the time you were 11–17 years old*?” From these responses, dummy variables were created to indicate SP in childhood (yes or no), SP in adolescence (yes or no) and persistence in SP in childhood and adolescence (“yes” to both questions or “no” to at least one of the questions). Additionally, information was collected regarding the number of modalities (“none”, “one modality” or “two or more modalities”), SP time in years (“no year”, “at least one year”, “at least two years”) and type of sport (“individual and collective sports”, “only collective sports”, “only individual sports” or “none”).

### Physical activity (PA) in adulthood

Participants were monitored by ActiGraph accelerometers (ActiGraph, Pensacola, FL), model wGT3X-BT for seven consecutive days. The researchers performed the placement and attachment of accelerometers on subjects, who were instructed to fix the device at the level of the right iliac crest and use it throughout the monitoring period, except during sleep, bathing and water activities. For the accelerometer initialization, the frequency of 30Hz was selected to record information. The ActiLife software version 6.13.3 was used for data reduction. Data were considered valid when subjects reached minimum of 480 minutes of daily use of the accelerometer in at least 4 days, being at least one valid day of the weekend. The non-use period was defined as an interval of at least 60 minutes of consecutive zero counts, with tolerance of one to two minutes between records of 1 and 100 counts [31]. Cutoff points for the magnitude vector of the ActiGraph and derivatives for adults were applied to estimate the daily minutes spent in PA of light, moderate, vigorous and moderate to vigorous intensity (MVPA) [32]. Therefore, data were reinstated for 60-second epochs.

### Sociodemographic variables

Sociodemographic variables gender, age (years) and self-reported socioeconomic level were analyzed. To classify the socioeconomic level, the Brazil Criteria of Economic Classification questionnaire was applied, developed by the Brazilian Association of Research Companies [33]. It was found that more than 91% of the sample were classified in classes A and B (high socioeconomic status).

### Statistical analysis

Data were initially entered and organized in Microsoft Excel spreadsheet (Windows®) and later processed and stored in the Statistical Package for the Social Sciences (SPSS for Windows Version 20.0). The Kolmogorov-Smirnov test was used to analyze data distribution. Frequency, mean and standard deviation values were used for sample characterization. Independent Student’s t or the Chi-square tests were used for comparisons between sexes.

Analysis by Generalized Estimation Equations (GEE) was used to establish comparisons between the different PA intensities in adulthood (light, moderate, vigorous, MVPA) according to SP specificities in childhood and adolescence (number of sports, type of sport and practice time) and SP in childhood and adolescence (both, any, none). For analyses by GEE, the probability of Gamma distribution was adopted for variables and, in the identification of differences between comparison groups, *post hoc* Bonferroni test was used. All analyses were stratified by sex and controlled by the total time of accelerometer use (minutes/day).

To verify the association between SP in childhood and adolescence and PA in adulthood, multiple linear regression analysis was used, stratified by sex. The different PA intensities (minutes/day) in adulthood were defined as dependent variables and transformed by logarithm base 10 to meet the assumption of data normality. The following independent variables were considered: SP in childhood (dichotomous), SP in adolescence (dichotomous) and persistence in SP in childhood and adolescence (dichotomous) transformed into dummy variables. The adjustment variable included in the analysis was total time of accelerometer use (minutes/day). Multicollinearity was verified, and the Variance Inflation Factor (VIF) values were low (VIF < 5) for all analyses, indicating that there is no collinearity between independent variables [34]. For all analyses, statistical significance adopted was *P* <0.05.

## Results

Of the 142 participants, 13 (8 male) subjects were excluded from the analyzes for not having valid accelerometry data. The descriptive characteristics of the sample are shown in Table 1. It is noteworthy that there was a statistically significant difference between sexes for all anthropometric variables. The SP frequency in childhood was considered higher in males when compared to females (*P* = 0.010), and this difference was not found for SP in adolescence. Regarding the daily time in each of PA intensities (light, moderate, vigorous and MVPA), no difference between sexes was identified.

**Table 1:** Descriptive characteristics of the sample (n = 129) by sex. Continuous values presented as mean ± standard deviation and frequency data in percentage values.

Variables	Males (n = 65)	Females (n = 64)	*P*
**Age (years)**	22.5 ± 1.6	22.3 ± 1.7	0.314
**Body mass (kg)**	76.7 ± 12.5	60.5 ± 10.9	<0.001
**Height (m)**	177.1 ± 7.2	164.8 ± 6.7	<0.001
**BMI (kg/m** ^**2**^ **)**	24.4 ± 3.2	22.2 ± 3.5	<0.001
**SP–childhood**			0.010
Yes (%)	90.8	73.4	
No (%)	9.2	26.6	
**SP–adolescence**			0.056
Yes (%)	89.2	76.6	
No (%)	10.8	23.4	
**PA in adulthood**			
Light PA (min/day)	812.4 ± 124.3	827.2 ± 140.1	0.528
Moderate PA (min/day)	31.9 ± 15.7	30.8 ± 17.1	0.685
Vigorous PA (min/day)	4.1 ± 4.4	3.6 ± 6.8	0.603
MVPA (min/day)	36.7 ± 19.2	35.0 ± 20.4	0.624

Note: BMI = body mass index; SP = sports participation; PA = physical activity; MVPA = moderate to vigorous physical activity; min/day = minutes of physical activity per day; *P* = statistical significance regarding the comparison between sexes using the independent Student t test and the Chi-square test.

Considering SP in youth, the most frequently mentioned sports modalities for males during childhood were: futsal (30%), soccer (20%) and swimming (20%), which did not change during adolescence. For girls, the most practiced sports in childhood were: swimming (32.5%), artistic gymnastics (19.5%) and volleyball (9%), being changed in adolescence to volleyball (19%), swimming (17%) and basketball (16%).

The daily averages of the different PA intensities in adulthood according to SP specificities in childhood and adolescence are shown in Table 2. For females, SP in childhood and adolescence resulted in a series of more favorable parameters in relation to PA in adulthood. The higher number of sports practiced in childhood presented longer time in vigorous PA (two or more = one ≠ none; *P* <0.05) and MVPA (two or more ≠ none; *P* <0.05). The same was observed with number of sports practiced in adolescence with MVPA (one ≠ none, *P* <0.05). The type of sport practiced in childhood presented longer time in vigorous PA (individual ≠ none, *P* <0.05) and MVPA (combination ≠ none; *P* <0.05). Subjects who practiced collective sports in adolescence spent more time in moderate PA and MVPA. Finally, SP time of at least one year in youth was related to longer time in light PA and vigorous PA in adulthood. In contrast, in males, all statistically significant differences found for PA in adulthood in relation to the number of sports in adolescence, type of sports in adolescence and practice time were configured as inverse, with an advantage for those who reported no SP.

**Table 2:** Physical activity in adulthood according to specificities of sports participation in childhood and adolescence by sex (n = 129). Values presented as mean and standard deviation ($\bar{X}$ ± SD).

Sports participation in childhood and adolescence	Physical activity in adulthood (minutes/day)							
	Light		Moderate		Vigorous		Moderate to Vigorous	
	Males ($\bar{X}$ ± SD)	Females ($\bar{X}$ ± SD)	Males ($\bar{X}$ ± SD)	Females ($\bar{X}$ ± SD)	Males ($\bar{X}$ ± SD)	Females ($\bar{X}$ ± SD)	Males ($\bar{X}$ ± SD)	Females ($\bar{X}$ ± SD)
**N. sports childhood**								
Two or more	806.6±133.4	870.9±176.1	29.1±14.1	30.0±11.4	3.7±3.9	6.3±10.2^a^	33.4±17.5	37.7±16.5
One	818.4±124.1	798.2±132.5	36.6±17.6	35.2±23.1	4.7±5.2	2.9±4.1	42.2±21.6	38.5±26.3^a^
None	818.2±81.2	813.2±78.0	27.6±11.1	25.1±10.5	3.6±3.8	0.9±0.8^b^	31.3±13.7	26.3±11.2^b^
**N. sports adolescence**								
Two or more	799.1±137.5	826.5±130.7	29.5±13.8	32.6±22.9	3.9±4.2ª	3.8±7.8	34.0±16.9	36.7±26.3^a^
One	829.4±114.2	822.8±142.1	34.3±18.8	32.6±15.5	3.5±4.8ª	4.5±7.5	38.3±23.3	38.1±19.4ª
None	823.3±87.9	836.9±155.6	36.3±13.3	24.7±10.6	7.3±2.7^b^	1.4±1.6	45.0±14.9	26.5±10.8^b^
**Type sport childhood**								
Combination	805.2±133.9	860.8±187.1	29.9±5.0	30.9±11.4	4.3±4.1	9.1±12.5	35.0±18.6	42.1±17.8^a^
Collective	819.6±129.3	869.1±219.2	36.1±17.6	37.5±12.3	4.1±5.0	2.4±2.3	40.8±21.6	40.2±13.4
Individual	805.8±118.6	811.0±128.2	26.3±10.3	32.7±22.3	3.8±4.2	2.9±3.9^a^	31.3±14.2	35.9±25.3
None	818.2±81.2	813.2±78.0	27.6±11.1	25.1±10.5	3.6±3.8	0.9±0.8^b^	31.3±13.7	26.3±11.2^b^
**Type sport adolescence**								
Combination	775.1±107.7	822.9±136.2	29.9±14.2	30.9±23.3	4.1±4.3	5.2±9.8	35.0±17.4	36.2±27.7
Collective	837.5±141.8	799.8±110.1	33.7±18.2	38.9±18.8ª	3.9±4.9	3.6±4.3	38.0±22.4	42.9±21.6ª
Individual	799.2±113.7	858.6±168.2	27.1±10.5	25.3±9.9^b^	2.3±2.7^a^	4.5±9.6	29.6±12.8	31.2±16.7
None	823.3±87.9	836.9±155.6	36.3±13.3	24.7±10.6^b^	7.3±2.7^b^	1.4±1.6	45.0±14.9	26.5±10.8^b^
**Practice time**								
At least two years	813.5±130	837.9±148.3ª	32.7±15.4	28.4±15.3^a^	3.8±4.4	3.9±7.7ª	37.2±19.1	33.3±19.2
At least one year	767.4±104.7^a^	818.9±107.4ª	26.0±18.6	36.5±21.5	4.1±3.9	3.1±3.4ª	30.7±22.3	39.8±24.4
None	875.7±84.8^b^	644.7±37.17^b^	36.1±11.7	38.4±6.2^b^	6.2±5.5	0.3±0.4^b^	43.1±14.4	38.7±6.7

Note: N. = number; Combination = practice of individual and collective sports; Collective = practice of collective sports; Individual = practice of individual sports; Different letters indicate P <0.05 in the intergroup comparison. All comparisons by GEE were controlled by total time of accelerometer use (min/day).

**Fig 2:**
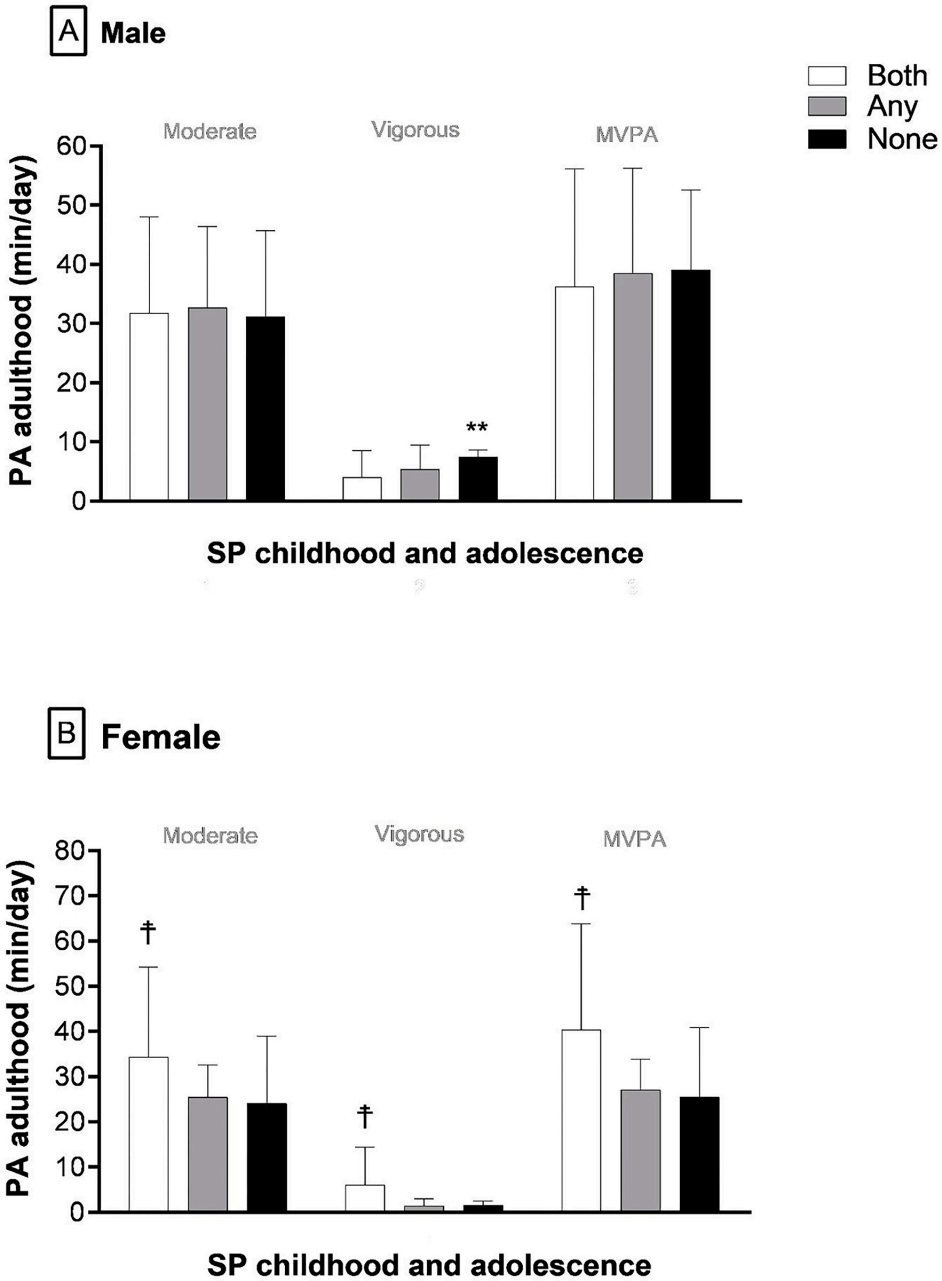
Comparison of physical activity at different intensities in adulthood according to sports participation in childhood and adolescence by sex (males n = 65; females n = 64). **Note:** Both = sports participation in childhood and adolescence; Any = sports participation only in one phase: childhood or adolescence; None = Did not participate in sports in childhood or adolescence. ☨ = P <0.05, both is different from any none; ** = P<0.05, none is different from any and both. All comparisons by GEE were controlled by the total time of accelerometer use (min/day).

Fig 2 illustrates analysis by GEE to verify association between different PA intensities in adulthood and SP in childhood and adolescence. For females, higher levels of moderate PA, vigorous PA and MVPA were identified among those who reported SP in both phases (childhood and adolescence) compared to those who reported SP in any phase (childhood or adolescence). However, only for vigorous PA, significant difference was identified in relation to the “none” group (reported no SP). For males, subjects in the group that did not report SP in both phases had more vigorous PA when compared to the SP group in childhood and adolescence (none > both).

The results of the analysis of association between SP in childhood and adolescence and practice of PA at different intensities in adulthood are shown in Table 3. SP in childhood, as well as persistence in SP (childhood and adolescence) were predictors of vigorous PA in adult females, that is, those who reported SP in childhood (β = 0.315; P = 0.016) and persistence in SP (β = 0.340; P = 0.010) showed higher levels of vigorous PA in adulthood. Additionally, SP in childhood and persistence in SP in females, including total time of accelerometer use in the model explained about 16% to 18% of vigorous PA in adulthood. The other significant relationships found between SP in childhood and persistence in SP with light PA for females and SP in adolescence with vigorous PA for males were considered to be inverse.

**Table 3:** Association between sports participation in childhood and adolescence and practice of physical activity at different intensities in adulthood by sex (n = 129).

PA in adulthood (minutes/day)	Males (n = 65)				Females (n = 64)			
	β (CI 95%)	β adjusted*	R^2^	*P*	β (CI 95%)	β adjusted*	R^2^	*P*
	**SP in childhood**				**SP in childhood**			
**Light PA**	-0.005 (-0.005 to 0.014)	-0.021	0.96	0.356	-0.009 (-0.001 to -0.018)	-0.060	0.95	**0.035**
**Moderate PA**	0.003 (-0.191 to 0.186)	0.003	0.09	0.978	0.081 (-0.238 to 0.076)	0.138	0.04	0.306
**Vigorous PA**	0.009 (-0.523 to 0.542)	0.005	0.02	0.972	0.418 (0.767; 0.068)	0.315	0.14	**0.020**
**MVPA**	0.005 (-0.211 to 0.201)	0.006	0.07	0.959	0.116 (-0.277 to 0.045)	0.179	0.02	0.153
	**SP in adolescence**				**SP in adolescence**			
**Light PA**	0.001 (-0.009 to 0.009)	0.000	0.96	0.987	-0.004 (-0.004 to 0.013)	-0.027	0.95	0.324
**Moderate PA**	-0.050 (-0.125 to 0.226)	-0.069	0.09	0.569	0.089 (-0.246 to 0.068)	0.146	0.03	0.261
**Vigorous PA**	-0.472 (-0.027 to -0.016)	-0.274	0.05	**0.038**	0.124 (-0.522 to 0.275)	0.086	0.05	0.536
**MVPA**	-0.100 (-0.090 to 0.291)	-0.128	0.09	0.297	0.111 (-0.272 to 0.051)	0.171	0.02	0.176
	**SP in childhood and adolescence**				**SP in childhood and adolescence**			
**Light PA**	-0.003 (-0.005 to 0.010)	-0.016	0.98	0.482	-0.008 (-0.000 to -0.015)	-0.054	0.95	0.056
**Moderate PA**	-0.022 (-0.121 to 0.165)	-0.037	0.09	0.760	0.089 (-0.228 to 0.050)	0.167	0.03	0.207
**Vigorous PA**	-0.222 (-0.164 to 0.608)	-0.150	0.01	0.254	0.439 (0.754 to 0.125)	0.364	0.18	**0.007**
**MVPA**	-0.041 (-0.115 to 0.197)	-0.063	0.08	0.600	0.128 (-0.270 to 0.014)	0.228	0.04	0.077

Note: PA = physical activity; SP = sports participation; MVPA = moderate to vigorous physical activity; CI = confidence interval.

* Multiple linear regression adjusted by the total time of use of the accelerometer (minutes/day), age and socioeconomic level. Bold values indicate statistical significance.

## Discussion

The main findings of the present study demonstrated that there was positive association between SP in childhood and adolescence and PA in adulthood only for females, in which SP in childhood and persistence in SP (childhood and adolescence) were predictors of vigorous PA. For males, significant association was observed between SP in adolescence and moderate PA and vigorous PA in adulthood; however, it was characterized in an inverse way. In addition, number of sports, type of sport and estimated SP time in childhood and adolescence were positively associated with vigorous PA and MVPA only for females (two or more sports in childhood; one sport in adolescence; collective sports in adolescence; at least one year of SP throughout childhood and adolescence).

Characteristics related to sport modalities most practiced in childhood and adolescence were also analyzed in this work. For males, the most cited sports modalities were soccer and futsal, both in childhood and adolescence. While for females, individual sports in childhood (swimming and artistic gymnastics) and volleyball in adolescence prevailed [25]. These results were similar to those obtained in a study conducted in Brazil [22], demonstrating that these modalities are quite culturally widespread in the country, as well as in a systematic review on the topic [7]. In addition, the work carried out by Azevedo et al. [22] also showed that individuals involved with PA in adolescence (represented by the practice of organized activities and sports) were more likely of being sufficiently active in adulthood, and this effect was greater for females, which corroborates the findings of the present study.

In this sense, more consistent results of the association between SP in childhood and adolescence and PA in adulthood, for females, were also verified in other studies [25]. Hirvensalo et al. [14] identified that females who practiced competitive or recreational sports at the age of 10–19 years were more likely of presenting high levels of PA in adulthood when compared to males; however, both showed statistically significant association. Likewise, a study with Finns followed from 14 to 31 years of age, found that the minimum frequency of one day per week of SP during adolescence for girls was already sufficient to increase levels of PA in adulthood against the frequency of twice a week for males, that is, it seems that the greatest effect of SP occurred for females [13]. More recently, two studies concluded that SP in youth could be more important among females than males for predicting physical activity in adulthood [25, 26].

Mäkelä et al. [15] investigated whether the number of sports practiced in adolescence would increase leisure levels of PA in adulthood and as in the present study, found association only for females (OR = 1.86 for three modalities and OR = 3.12 for five modalities). In addition, Sylvester et al. [13] concluded that practicing a variety of sports during adolescence is positively associated with moderate-vigorous exercise during adulthood, and this experience may, in part, explain this relationship.

Sex differences in PA outcomes are complex, therefore, some pathways suggested in literature could explain the results of the present study. Boys have been considered more active than girls [1, 35, 36], and also have greater stability in these behaviors when compared to females [8, 9]. Perhaps, this explains the fact that SP in childhood and adolescence had greater effect on girls in the present study, being characterized as a predictor of PA in adulthood, particularly vigorous PA. Changes inherent to the transition from adolescence to adulthood (living alone, employment, marriage, pregnancy, among others) must also be taken into account, which are different in some aspects between sexes [12] and can impact in a different way on observed associations.

The associations found in the present study for males demonstrated an inverse relationship between PS in adolescence and PA in adulthood, which have not been confirmed by literature findings to date [7]. Information on the behavior of PA in youth may, in part, explain these results. A systematic review developed by Dumith et al. [37] investigated changes in PA in young people and the authors identified greater declines in PA for males during adolescence (13–16 years), while for females, it occurred between childhood and adolescence (9–12 years). Probably, boys who stopped engaging in sports at this phase (no SP) returned to practice systematized physical activities in adulthood and showed higher values of moderate and vigorous PA indicators when compared to those who reported SP in adolescence.

In addition, another aspect that should be highlighted about the complex behavior of PA throughout life is the fact that the adolescence phase is characterized by great influence from the group to which the adolescent is inserted (friends, school class), being characterized as one of the main barriers (lack of company, friends live far away) for the practice of PA at this phase of life [38]. However, upon reaching adulthood, after this transition phase, boys who did not report SP in adolescence return to engage in systematized practices, since the group’s attitudes do not exert much influence on behaviors adopted at this time. Therefore, inverse associations can be found, as observed in the present study.

This work was one of the pioneers in Brazil to study the theme of SP in childhood and adolescence in a specific and detailed way, associating it with PA outcomes in adulthood objectively assessed by accelerometry. This fact allowed analyses to take into account the different PA intensities (light, moderate, vigorous and MVPA), with additional stratification by sex, which was an important strength of this work. However, limitations should also be considered, such as the high frequency of SP reporting in the entire sample, both in childhood and in adolescence, which may impact the results found in some way. However, in an attempt to minimize this bias, comparison analysis was used considering this uneven distribution between groups, which was the analysis by GEE. Additionally, limitations inherent to SP evaluation retrospectively performed should also be considered. Therefore, it was considered only the practice of sports that characterize SP, not taking into account participation in physical exercises, since the sport has advantages related to its recall, particularly when practice was performed for at least one consecutive year, as considered in this study. Finally, the lack of information about SP in adulthood was also considered a limitation, especially when it comes to discussing the findings of the present study.

In view of the above, important results have been obtained in the present study regarding the association between SP in young people, its specificities, and the practice of PA at different intensities in adulthood. However, there are few longitudinal studies in literature regarding the impacts of SP on children and adolescents, their interaction with maturation, type of sport [20] and training overload [39] and how these relationships are presented throughout the life of these young sports practitioners, whether athlete or non-athlete.

## Conclusion

It could be concluded that sports practice in childhood and persistence in SP were considered predictors of vigorous PA in adult females. In addition, playing two or more sports in childhood, one sport in adolescence, collective sports in adolescence and at least one year of SP throughout childhood and adolescence, can provide higher levels of vigorous and MVPA in adult females.

### Practical implications

- In view of the current and alarming epidemic of physical inactivity, the identification of characteristics of sports participation (SP) in youth that could predict higher levels of physical activity (PA) in adulthood is very promising.
- Our findings were able to demonstrate interesting results of the relation of SP in childhood and adolescence with increased levels of vigorous PA and MVPA in adult females.
- However, this study represents the beginning of research on this specific theme in Brazil, a developing country with continental dimensions and unique social, economic, and cultural characteristics.

## Supporting information

S1 Checklist STROBE statement—checklist of items that should be included in reports of observational studies. (PDF)

S1 File Excel file. (XLSX)
